# Supplementary material for: Are carbon emissions trading and green financial instruments synergistic? -Comprehensive quantitative research based on content analysis
Source: PLoS One. 2024 Mar 7;19(3):e0298601. doi: 10.1371/journal.pone.0298601 (PMC10919601; doi:10.1371/journal.pone.0298601)
Supplement: S1 Data — (ZIP) [file pone.0298601.s001.zip › Data/地级市Z_F工作报告.zip!/╕ú╜¿/╧├├┼╩╨/└┤╫╘╣1⁄2╓┌║┼í╛┬φ┐╦╩2╛▌═°í┐_2007.docx]

各位代表：

　　现在，我代表市人民政府向大会作政府工作报告，请予审议，并请市政协委员和各位列席人员提出意见。

　　一、2006年和本届政府的工作回顾

　　2006年，是厦门经济特区建设二十五周年，我们在中共厦门市委的领导下，高举邓小平理论和“三个代表”重要思想伟大旗帜，全面贯彻落实科学发展观，努力构建社会主义和谐社会，积极融入海峡西岸经济区建设，扎实推进新一轮跨越式发展。经过全市人民的共同努力，完成了市十二届人大四次会议确定的各项任务，实施“十一五”规划有了良好开局。全年地区生产总值1162．4亿元，增长16．7%；工业总产值2443．8亿元，增长20．4%；全社会固定资产投资662．1亿元，增长66．5%；财政总收入275．2亿元，增长31．2%，其中地方级财政收入143．9亿元，增长38．6%；城镇居民人均可支配收入18513元，农民人均纯收入6868元，分别增长12．9%和10．2%；城镇登记失业率3．74%，居民消费价格指数100．85，人口出生率10．8‰。

　　这一年，中共中央总书记、国家主席胡锦涛，中共中央政治局常委、全国政协主席贾庆林先后视察厦门，充分肯定了厦门经济特区发展的巨大成就，给予我市干部群众极大鼓舞，全市上下形成了推动工作、推动建设、推动发展的强大合力，形成了持续推进新一轮跨越式发展的如虹气势、强劲态势和风清气正、人和业兴的良好趋势。固定资产投资、利用内外资、第三产业、财政收入、城乡居民收入等主要经济指标是近年来增长最好最快的一年。集装箱吞吐量突破400万标箱，跻身世界20强。规模浩大的环东海域综合整治建设工程全面启动，“金包银”工程和社会保障性住房建设成为全国的创举。成功举办第四届世界合唱比赛，国家主席胡锦涛亲自发来贺信。第十届中国国际投资贸易洽谈会和首届中国（厦门）名牌产品国际采购交易会获得圆满成功。荣获世界银行评选的投资环境中国大陆金牌城市，入选全国最受农民工欢迎的十大城市。

　　一年来，我们主要做了以下工作：

　　（一）加快落实工业优先发展战略。火炬（翔安）产业区、同安工业集中区、集美机械工业集中区、海沧石化工业集中区、出口加工区等加快建设，433万平方米标准厂房相继建成，300多家企业入驻园区，载体的吸附器和启动器效应开始显现。厦华电子工业园、龙欣利照明（T5节能灯）等项目相继开工，友达光电、太古飞机维修五期等项目建设加快，大金龙实现易地搬迁，戴尔二期、宸鸿科技、联想移动、明达光电等项目竣工投产。大小金龙获得汽车底盘生产资质，厦门成为国家级汽车及零部件出口基地。增拨财政资金1亿元，集中扶持台湾科技企业育成中心、钨材料工程技术研发中心、半导体照明产品检测与营销中心等十大科技平台和公共技术服务平台，与中国科学院共建城市环境研究所，厦门知识产权产业化基地正式开园，全市发明专利授权量增长43．2%。工业结构进一步优化，产业素质明显提高，重点高新技术工业企业产值占规模以上工业总产值55．9%，新增2个中国驰名商标、3个中国名牌产品、3个出口免验产品和12个国家免检产品。

　　（二）全力推进基础设施建设。对接周边的福厦、龙厦铁路全面动工，连接岛内外的翔安海底隧道、杏林大桥、集美大桥工程加快推进，同安大桥、集美大道、疏港路改造一期全线通车，成功大道、环岛干道、环东海域滨海大道等城市主干道建设进展顺利。东渡航道扩建工程完工，嵩屿港区一期和海沧港区在建的十个泊位取得新进展，海沧500千伏、翔安220千伏变电工程投产。建成一批公交场站，第二、三期LED夜景工程投入使用。新增公共绿地507公顷，植树造林2．1万亩，天竺山森林公园建成开园。建成筼筜、翔安污水厂和鼓浪屿三个污水处理站，完成同安官浔溪河道整治。全年固定资产投资投入大、项目多、后劲足、效果好，对经济增长贡献率达45%。

　　（三）着力增强中心城市服务功能。厦门港实现一体化管理，现代物流园区初具规模。杏北新城、翔安东方新城的住宅区相继落成，中华、厦港片区旧城改造稳步推进，软件产业基地、五缘湾、观音山国际商务营运中心和园博园建设加快。微软、IBM、西基等著名软件企业进入厦门，软件业产值翻番。翔鹭国际、海悦山庄等一批高星级酒店开业，国际旅游客运码头、国际会议中心等项目建设顺利推进。接待海内外游客1800多万人次，其中海外游客突破百万人次。成功举办全国“植保双交会”等大型展会，厦门石材展成为亚洲最大的石材专业展会。明发商业广场、名汇铜锣湾广场等大型购物中心投入运营，国美、苏宁等部分大卖场向岛外拓展。美国首都银行、中英人寿、都邦财险等金融机构落户厦门，全市金融机构本外币存款余额突破2000亿元。完成上市公司股权分置改革。港口物流、旅游会展、金融商贸、信息咨询等中心城市服务功能明显提升。

　　（四）努力提高改革开放水平。完善征地拆迁政策，理顺市对区管理体制，整合重组十家市直管国企集团及十三家委托市直部门和开发区管理的国企集团，绿泉实业整体转让，燃气公司引进战略投资者。招商引资创历史最高，合同利用外资23．1亿美元，增长78．5%，实际利用外资9．6亿美元，增长34．9%；协议内资382．8亿元，增长32．0%，实际利用内资162．4亿元，增长40．6%。民营经济总量壮大、活力增强。积极适应人民币升值和出口退税政策调整，财政增投1亿元扶持扩大出口，东渡、海沧和高崎机场三个海空口岸实行七天工作制，通关条件进一步改善，外贸进出口总额327．9亿美元，出口额突破200亿美元。

　　（五）不断拓展对台交流合作。全年合同利用台资（含第三地）6．9亿美元，实际利用台资3．7亿美元，分别增长36．5%和19%，友达光电、中华映管、新晶电等一批知名台企落户厦门。五通海空联运码头基本建成，厦金海空联运由“华东一条龙”升级为“两岸一票通”，厦金班船由每天12班增至20班，年旅客吞吐量突破60万人次。开办厦金航线包裹业务，顺利完成两岸春节、中秋节包机直航。厦门台湾水果销售集散中心正式挂牌，台湾水果进口量增长一倍多。开设台生班，取消台生借读费，首办台湾地区专业人才暨大学毕业生大陆就业洽谈会。成功举办第十届“台交会”、海峡两岸农业合作成果展览暨项目推介会、央视中秋晚会、海峡两岸图书交易会、闽南语原创歌曲大奖赛、首届两岸青年联欢节等重大活动，爱乐乐团、小白鹭民间舞团、歌仔戏剧团等赴台演出，厦门在对台交流中的区位优势日益凸显。

　　（六）全面启动社会主义新农村建设。制定出台《新农村建设五年规划纲要》，完成73个行政村村庄规划，首批20个旧村改造和新村建设试点进展顺利，通行政村道路硬化全部完成，公交通达率86%，农村自来水入户率90%，绿化、路灯、排污、垃圾处理等市政配套引入农村，提前一年完成156个农村义务教育和公共卫生项目。“金包银”、“金包金”工程开工建设面积52万平方米，部分已经交付使用并开始收益。支持18个村利用农民征地款集资入股建设通用厂房、外口公寓等项目，3万多被征地农民有了稳定持久的收入。农村1．2万未升学初高中毕业生进入中等职业学校就读。推动31个经济薄弱村实施挂钩帮扶项目，许庄、大帽山农场等一批移民造福工程进展顺利。市级农业产业化龙头企业增至35家，产值增长10．7%，新增订单农业1．6万亩，吸纳本地农民工2．7万人。全年完成农村劳动力培训7000多人，转移就业2．42万人。全市农民人均纯收入和增幅保持全省第一。

　　（七）加快发展社会事业。全市财政用于教育、卫生、文化、体育等社会事业支出31．3亿元，增长32．5%。整合教育资源，城乡中小学办学条件普遍改善。集美、翔安文教区建设继续推进，厦门理工学院、华侨大学厦门校区、职业教育中心校等工程进展顺利，厦门医高专获批正式组建。中医院、第三医院、疾控中心、紧急救援中心完成迁建并投入使用，第一医院综合病房楼、第二医院二期、中山医院外科大楼等基本完工。开展“关爱女孩”行动，加强人口和计生工作，计划生育政策符合率96．6%。文化艺术中心建成开馆，小白鹭艺术中心、闽南戏曲艺术剧院建设进展顺利。歌仔戏、高甲戏、南音、漆线雕、答嘴鼓等荣登首批国家级非物质文化遗产名录。歌仔戏《邵江海》获全国优秀地方剧目展演一等奖。嘉庚体育馆、厦门足球训练基地等投入运营，城乡体育设施进一步改善，在全国体育大会和省运会上取得较好成绩。与此同时，侨务外事、人事编制、气象防震、方志档案等工作得到加强。

　　（八）积极构建和谐社会。全年新增就业17．8万人，在全国率先将建筑、矿山等高风险行业的农民工纳入工伤保险，率先出台了包括老年人、未成年人在内的全民医保，率先建设社会保障性住房解决中低收入家庭的住房困难。继续实施农村部分计划生育家庭奖励扶助制度，提高了最低生活保障标准和最低工资标准。被征地农民参加基本养老保险超过3万人，已有1．65万人按月领取养老金。创造条件让农民工逐步享受市民待遇，免费接受职业培训、岗前培训和就业服务，对民办学校接纳农民工子女就读的给予财政支持，取消了农村义务教育学杂费和进城农民工子女借读费。努力解决好广大群众关心的看病就医问题，开设惠民门诊、病房，全面开展免费婚检和优生检测，为家庭经济困难妇女免费提供妇科疾病检查，设立建筑工人人身意外伤害急救“绿色通道”，全年平均个人门诊、住院医疗费用保持零增长。深入开展“平安厦门”建设，建立城市治安报警监控系统，全面落实群防群治，依法严厉打击严重刑事犯罪。精心实施“五五普法”规划，增强公民的法律意识。进一步畅通信访渠道，完善多元化纠纷解决机制，人民内部矛盾纠纷得到有效化解，社会治安满意率保持全省第一。加强安全生产专项整治，编制修订各类突发事件应急预案，成功组织10万人防灾应急演练。健全非典、禽流感等疾病预防控制体系，全面启动重点污染源自动监控系统和洪水预警系统，建立全国首个灾害应急救援中心，实施交通改善工程，各类事故下降47．1%。十件为民办实事项目全部完成。人防海防、民兵预备役、民族宗教、妇女儿童、老龄、残疾人等工作取得新成绩，社区在构建和谐社会中发挥了积极的作用。

　　（九）不断加强政府自身建设。推进依法行政，认真执行《公务员法》和《行政许可法》，健全政府重大决策及规范性文件法律审查制度，完成行政执法依据清理，出台和修订了4件政府规章。深化行政审批制度改革，积极推行“一站式服务，一个窗口对外”的审批方式，区域性集中办事场所增至15个，104个项目实现网上审批，重大项目实现就地审批，行风建设取得成效，群众对服务窗口的满意率进一步提高，政府管理竞争力在全国200多个大中城市中排名第三。全面推行以公共资源配置市场化改革为核心的“阳光工程”，推出市场化配置项目64项。加强审计监察工作，在工程承包、土地出让、产权交易等六大重点领域治理商业贿赂，对重大项目征地拆迁实行全程跟踪监督，全市社保基金通过国家审计安全完整。坚持向人大及其常委会报告工作，依法接受人大法律监督、工作监督，自觉接受政协民主监督，认真办理人大代表议案、建议239件，政协提案470件，办结率达100%，满意率分别达96．3%和96．1%。大力推行政务公开，充分发挥“市长热线”、市政府新闻发布会与政府网站作用，加强同民主党派、工商联和社会团体的联系，广泛听取群众意见。

　　各位代表！

　　根据中央部署和省人大常委会决定，本届政府提前一年换届。

　　过去的四年，是振奋精神、迎难而上，城市综合竞争力不断提高的四年。我们有效抗御了台风暴雨、非典、禽流感等灾害，克服了用地短缺、煤电油运紧张等困难，在遭遇挫折中奋起，在战胜困难中前进，积极适应中国加入WTO和国家重大政策调整，启动新一轮跨越式发展，实现了经济社会又好又快发展，地区生产总值由648亿元提高到1162亿元，工业总产值由1112亿元提高到2444亿元，财政总收入由126亿元提高到275亿元，年均分别增长16．4%、23．4%和22．2%。在全国200个大中城市的综合竞争力排名从第十七位跃居第九位。

　　过去的四年，是科学发展、调整提升，质量、效益与速度同步增长的四年。三次产业结构由3．4∶53．1∶43．5调整为1．6∶54．0∶44．4，重点高新技术工业企业产值占规模以上工业总产值比重由51．3%提高到55．9%。全市财政总收入占生产总值比重由19．5%提高到23．7%，常住人口人均生产总值突破6200美元，每平方公里土地面积创造生产总值7407万元，位居全国前列。厦门以占全省6．5%的常住人口和1．3%的土地，创造出占全省15．5%的生产总值、26．4%的财政收入和52．3%的外贸进出口总额。

　　过去的四年，是牢记特区使命、先行先试，改革开放取得新突破的四年。实施行政区划调整，完善市区两级财权、事权，大幅压缩建设项目审批时限，实行经评审的最低价中标和业主招标。推进事业单位改革改制，整合重组国有资产投资公司和授权经营集团，实行有限行政资源公开“招拍挂”，提高市场化办展、办赛水平，各项改革稳步推进，社会主义市场经济体制更加完善。四年实际利用外资27亿美元，实际利用内资近300亿元，年进出口总额由152亿美元增加到328亿美元，港口集装箱年吞吐量从175万标箱迅速增长到401万标箱，空港旅客年吞吐量由426万人次增加到750万人次。新增国际友城3个，举办大型国际会议与展览525场次，国际知名度不断提高。

　　过去的四年，是依托“五缘”、拓展“六求”，对台优势进一步凸显的四年。积极承接台湾优势产业转移，累计引进台资（含第三地）项目643个，合同利用台资16．7亿美元，对台贸易额59．7亿美元。厦门与高雄港集装箱试点直航、厦金直航、厦金旅游、厦门航点两岸包机直航成效显著，厦台农业合作、会展旅游、文化交流取得新突破，开办五年期台胞证办理中心，民间交往进一步密切，经厦门口岸来往的台胞从32．5万人次上升为82．4万人次，在厦购房置业、求学就医的台胞成倍增长，两岸人才交流平台建设初见成效，台胞权益得到有力保障，对台交流合作基地作用更加突出。

　　过去的四年，是以人为本、关注民生，和谐发展的四年。社会事业和民生保障投入最大、城乡居民受益最多，财政累计投入178亿元，新建扩建了一大批社会事业项目，教育卫生和文化体育设施得到极大改善，城乡社会保障水平领先全国，最低工资标准和最低生活保障线分别提高44．4%和23．0%，全民医保和农村新型合作医疗基本建立，新增就业69．2万人，城镇居民人均可支配收入和农民人均纯收入年均递增12．0%和9．8%，城镇居民恩格尔系数由41．3%下降到35．5%，城镇居民人均住房使用面积提高到22．9平方米，农民人均住房面积超过50平方米， 人均公共绿地面积13．53平方米，成功整治西海域，生态文明建设走在全国前列，获得“联合国人居奖”和“东亚海岸带政府管理杰出成就金奖”，荣获首批“全国文明城市”称号和“全国双拥模范城市”五连冠，厦门以文明、温馨、宜居享誉海内外。

　　以上成绩的取得，是在中共中央、国务院和中共福建省委、省政府及中共厦门市委的正确领导下，在各级人大及各位代表的依法监督和有力支持下，在各级政协及各位委员的民主监督和积极参与下，全市干部群众齐心协力、艰苦奋斗的结果。在此，我谨代表市人民政府，向全市人民，向各民主党派和工商联、人民团体、各界爱国人士，向离退休的老同志，向中央和省驻厦单位，向驻厦人民解放军、武警官兵和公安干警，向来厦的所有投资者和外来劳动者，向所有关心支持厦门的港澳同胞、台湾同胞、海外侨胞和国际友人，表示衷心的感谢和崇高的敬意！

　　各位代表！

　　回顾过去，成绩来之不易，经验弥足珍贵。四年的政府工作给我们留下深刻体会：必须坚持以科学发展观统领经济社会发展全局。坚持把发展作为第一要务，坚持以经济建设为中心，以发展工业为重点，通过工业的快速发展，壮大第三产业，提升第一产业，从而不断促进经济结构优化升级，推动经济社会又好又快发展。必须十分讲究发展的策略和方法。坚持先谋后动，谋事力成；坚持有所为，有所不为；坚持尽力而为，量力而行；坚持调动一切积极因素。必须坚定不移地推进改革开放。坚持解放思想、实事求是、与时俱进，不失时机地推进各项体制改革和制度机制创新，创造性地开展工作，始终保持经济社会发展的强大动力和活力。必须积极发挥对台优势。依托“五缘”，强化“六求”，积极融入海峡西岸经济区建设，促进海峡两岸经济文化交流，努力实现“一个作用、两个历史性贡献”。必须坚持执政为民，持续推进和谐社会建设。把人民利益作为一切工作的出发点和落脚点，下大力气解决人民群众最关心、最直接、最现实的问题，让广大群众受益，让各方面共享发展成果，持久地形成推动发展的整体优势和强大合力。

　　政府工作还存在一些亟待解决的问题，一是落实科学发展观、构建和谐社会的能力和民主科学决策水平有待进一步提高，对经济社会发展的深层次问题研究不够，解决实际问题和复杂矛盾的办法不够多，有时候存在办事效率低、行政成本高的问题，领导方式、工作方法等方面与新形势新任务的要求还不相适应。二是我们还处于并将长期处于社会主义初级阶段，经济社会发展水平与人民群众的期望和需求还存在较大差距，在住房、教育、医疗等方面还难以满足人民群众日益增长的需要，被征地农民转移就业任务还比较繁重。三是政府职能转变相对滞后，社会管理还存在薄弱环节，城市交通拥堵现象比较严重，社会治安面临新的严峻形势，影响安定和稳定的现象还时有发生，应对和化解矛盾问题的机制不够健全。同时，还面临一些制约经济社会发展的难题，主要是：经济总量偏小，经济腹地、市场空间拓展难度大，中心城市的辐射带动功能还较弱；创新型人才和高技能人才偏少，自主创新能力不足；改革的任务还很艰巨，一些体制性障碍亟待破解。对这些问题，我们要有清醒的认识，认真听取广大市民的意见建议，虚心学习其他城市的先进经验，切实加以解决。

　　二、今后五年工作展望和2007年工作建议

　　今后五年是我市加快发展的关键时期，面对国际间、城市间的激烈竞争，面对资源、环境、市场等方面的压力，我们要认真落实胡锦涛总书记视察福建、厦门的讲话精神，珍惜当前厦门大好的发展形势、依托厦门坚实的发展基础，紧紧抓住国家鼓励东部地区率先发展、支持海峡西岸加快发展的重大历史机遇，进一步解放思想，实事求是，善于用宽广的视野、战略的思维和大局的意识来谋划厦门的发展。

　　今后五年政府工作的总体要求是：坚持以邓小平理论和“三个代表”重要思想为指导，认真贯彻党的十六大和即将召开的十七大精神，认真贯彻省第八次党代会和市第十次党代会精神，牢固树立和认真落实科学发展观，积极构建社会主义和谐社会，全面落实省委关于建设海峡西岸经济区的总体部署，把“重在持续、重在提升、重在运作、重在实效”的实践要领贯穿于各项工作中，着力推进改革开放，坚持优先发展工业，大力发展第三产业，继续重视第一产业，实现结构调整和产业升级，提高经济增长质量，扩张经济总量，又好又快地推进新一轮跨越式发展，努力发挥厦门经济特区“带头带动、示范表率、促进引领、桥梁纽带”作用，努力成为以科学发展观统领经济社会发展全局的示范和榜样，努力成为构建社会主义和谐社会的示范和榜样。主要目标任务是：

　　—— 全面完成“十一五”规划目标

　　力争实现四个翻番，到2010年，确保地区生产总值、工业总产值、财政总收入比2005年翻一番，力争人均生产总值比2005年翻一番。综合能耗保持国内领先水平。构筑四大体系，即城市产业支撑体系、城市基础设施体系、城市公共信息平台体系、城市制度创新体系。力争实现三个协调发展，即速度、质量、效益相协调，消费、投资、出口相协调，人口、资源、环境相协调。在此基础上，认真制定并开始实施“十二五”规划，为“十二五”开好局，起好步。

　　—— 扎实推进中心城市建设

　　基本建成海峡西岸强大的先进制造业基地、航运物流中心、旅游商贸中心、文化教育中心和对台交流合作前沿平台，中心城市的辐射带动能力明显增强。岛外形成100平方公里设施完善、环境优美的新城区，城市建成区面积扩大到230平方公里，城市服务功能比较完善，现代化、国际化、信息化水平显著提高，建设现代化港口风景旅游城市和海峡西岸重要中心城市的目标基本实现。

　　—— 加快构建和谐社会

　　争取率先跨入宽裕型小康社会，人民生活更加殷实，到2010年城镇居民人均可支配收入和农民人均纯收入分别达到2．5万元和1万元左右，城镇登记失业率控制在4%以内。高水平、高质量普及高中阶段教育，常住人口千人医生数由2．15个上升到3．2个，实现城乡困难群众居有其屋、病有所医，低保和社保水平居全国前列。巩固和扩大“平安厦门”成果，群众的安全感不断提高。环境更加优美，大气质量保持优良水平，主要污染物排放总量控制在国家规定范围内，生活垃圾无害化处理率95%，环境保护和防灾减灾能力进一步提高，资源节约型、环境友好型城市建设取得明显成效。政府廉洁高效，社会民主进步，市民综合素质和城市文明程度进一步提高。

　　—— 大力加强对台交流合作

　　深入落实对台工作方针，认真履行中央赋予厦门的特殊使命，先行先试，力争扩大台商投资区，加强厦台产业对接，实现两岸经济技术交流合作向更大范围、更宽领域、更高水平发展。充分发挥闽南文化的优势，提升两岸民间文化交流活动层次，进一步拓宽海上直航功能，促进两岸全面、直接、双向“三通”，把厦门建成海峡两岸“产业投资、物流商贸、农业合作、人员往来、展会交流、文化交流”的重要前沿平台，为促进祖国统一大业作出更大贡献。

　　各位代表！

　　2007年是新一轮跨越式发展的重要一年，也是实现“十一五”目标的关键一年，主要预期目标是：地区生产总值增长16%，万元生产总值能耗降低2．52%，地方级财政收入增长23%，城镇居民人均可支配收入和农民人均纯收入分别增长10%和11%，城镇登记失业率控制在4%以内，居民消费价格指数103左右，人口出生率控制在11‰以内。在实际工作中要加倍努力，力争完成得更好。

　　政府主要工作是：

　　（一）优先发展现代工业，建设海峡西岸强大的先进制造业基地

　　着力抓好工业集中区建设。在五大工业集中区、出口加工区和环东海域开发地带新建一批标准厂房，力争基本竣工并完成招商。要十分重视海内外高新技术企业向厦门集聚、向工业集中区集聚，引导同类和关联产业项目向园区集聚，完善水、电、通讯等基础设施，完善原辅材料经销、金融保险等配套，完善商业网点、餐饮场所、文化娱乐设施，尽快开通公交车，搞好环境卫生和社会治安。通过劳务协作、委培代训等方式吸纳大批技术工人。推动友达光电、金龙汽车配套项目等竣工投产，加速推进华映工业园、厦华电子工业园等项目，开工建设华诚纺纤、金保利光电、冠捷电子等一批项目。加强对工业企业的跟踪服务，确保产值效益稳定增长。

　　积极推动科技创新。加快国家创新型城市建设，建立以企业为主体、市场为导向、产学研结合的技术创新体系，完善自主创新激励机制，落实鼓励创新的财税政策、金融政策和政府采购制度。鼓励风险投资，积极培育创新型中小企业。更加重视鼓励更多内外资企业在厦门设立研究开发中心，更加重视国内外先进技术的引进消化吸收再创新，更加重视具有技术关联性和产业带动性的集成创新。鼓励企业增加技改投入，应用高新技术和先进适用技术改造提升传统产业。加强科技研发，继续安排重大科技项目资金1亿元，重点支持科技创新公共平台建设，扶持拥有自主知识产权的重大产业科技项目，启动环东海域厦门科学园区等载体建设，争取设立厦门国家海洋科学研究中心。继续扶持视听通讯和钨材料等优势特色产业，重点培育光电子、软件、生物与新医药、仪器仪表等新兴产业，争取创建国家电力电工特色产业基地。争创国家知识产权示范城市，创造更多拥有自主知识产权的高新技术产品。深入实施名牌带动战略，提升厦门品牌的知名度。

　　做好资源节约工作。全面推进新一轮土地利用总体规划修编，科学编制用地计划，挖掘存量，集约节约用地，做好工业用地“招拍挂”工作，规范国有土地使用权出让收支管理，更新城镇基准地价。积极发展循环经济，建立投资项目节能评估审查制度，建设热电联产等百个节能示范项目，推广使用清洁能源和先进设备。深入开展环境友好企业、环境友好工程创建活动，推进物料厂际循环，减少废弃物最终排放，力争工业用水循环利用率达到92%以上。

　　（二）全面落实项目带动战略，着力推进中心城市建设

　　加快重点工程建设。全年安排固定资产投资850亿元，全面推进城市综合交通体系建设，开工建设厦漳跨海大桥、BRT快速公交系统，推进集美大桥、翔安海底隧道、成功大道等项目建设。全面构建集疏运体系，做好福厦、龙厦铁路施工保障，开工建设厦深铁路和厦门西客站综合交通枢纽，深入开展利用国铁开通城市列车方案研究，推进厦门—安溪城际快速路建设，做好厦成高速公路厦门段前期工作，加快建设进岛电力第四通道。继续推进海沧、集美、同安、翔安新城区和五缘湾片区建设，精心组织好环东海域和湖边水库综合整治各个环节和项目的施工，启动杏林湾新城区建设和马銮湾综合整治建设，做好厦港片区等旧城改造，基本建成观音山国际商务营运中心区一期工程。

　　提高城市建设管理水平。启动数字化城市管理工程。开展交通综合改善工作，缓解城市交通拥堵。提高仙岳山、东坪山等一批山体公园建设水平，做好天竺山森林公园、万石植物园等项目扩建改造工作，新增绿地600公顷，林相改造1135亩。继续推进LED夜景工程建设，建立健全夜景工程维护管理机制。加大力度治理汽车尾气、工业废气、工地扬尘污染，启动建设有毒有害危险废弃物综合处理厂，加快东部供水项目建设，推进北溪引水左干渠龙海段综合整治，新建海沧水厂、翔安水厂，扩建同安水厂，推进莲花水库和长泰枋洋第二水源前期工作，全面展开厦门岛排涝系统工程建设。力争两年内基本建立全市村镇生活垃圾管理系统，畜禽养殖场生态零排放达到90%以上，加大海洋环境保护和生态修复力度，继续开展海洋环境综合整治，通过区域合作和污染防治，减少九龙江入海污染物，建设红树林湿地、人造沙滩；建立健全厦门海洋环境监测网络、应急预警系统和海洋执法监控系统，全面提升海洋综合管理水平。

　　提升城市服务功能。加快发展生产性、商业性和消费性现代服务业。推动厦门港通关一体化，力争实现“同港同策”。推进现代物流园区和前场铁路特大型货场建设，加快建设厦门港十万吨级航道三期、海沧航道二期和东渡港区现代码头，支持开辟新航线，大力发展国际中转业务，推进区域通关协作，努力拓展物流腹地，创造条件开通厦门至南昌的集装箱班列，逐步确立厦门港作为内陆省市的重要出海通道。开展第二机场的选址论证工作，争取开通厦门至欧美的客运航线，扩大海铁、陆空、海空联运。办好“园博会”和“厦门国际海洋周”，进一步扩大投洽会、台交会的影响力。力促瑞景、加州商业广场建成并投入使用，推进SM二期、世贸商城二期建设，稳步推进“农改超”步伐，支持沃尔玛、好又多等商业企业向岛外拓展，促进岛外商业繁荣。通过特色商业街区的完善、旅游休闲设施建设以及重大节庆、展会活动，打造会展、旅游、商贸互动平台。提升旅游口岸服务功能，加大旅游公共服务设施投入，推进一批高星级酒店建设，发展邮轮经济，争创全国最佳旅游城市。大力吸引国内外企业来厦设立销售、研发、结算中心。出台鼓励措施，着力引进国内外金融机构，完成城市商业银行资产重组和增资扩股工作，鼓励和支持一批优质企业改制上市，推动金融创新。积极引进境内外知名中介服务机构，不断提高我市服务业的整体水平。

　　（三）全力推进新农村建设，让广大农民共享特区发展成果

　　千方百计增加农民收入。重点扶持高科技种苗业和高附加值农产品加工销售业，扩大订单农业规模，扶持农业产业化龙头企业。抓紧“金包银”项目的招商和招租工作，完善项目分配和运行管理机制。做好被征地农民和退养渔民转岗培训，鼓励和引导他们利用补偿款集资建设股份化项目。继续扶持农村未升学初高中毕业生进入中等职业技术学校学习。加大财政转移支付力度，扶持老区、边远村庄发展，启动新一轮挂钩帮扶经济薄弱村工作。

　　加快推进农村城市化。完成全市所有村庄的建设规划，在完成首批20个试点村项目建设的基础上，新启动25个重点村的旧村改造和新村建设。继续做好许庄、大帽山农场移民造福工程，推动莲花水库上游、汀溪大麦畲自然村等重点水源保护区和偏远山区农民向中心城镇搬迁。在岛外城镇、工业区、公路主干道周边和环东海域等重点镇村，加快实施“镇改街”、“村改居”，并在管理体制、社会保障、社区建设、公共服务配套等方面与城市社区接轨，逐步实现城乡一体化管理。

　　提高农村公共服务水平。完善农村电网、路网，加快通自然村的公路建设，提高农村自来水普及率、有线电视入户率和公交客运通达率。全面开展水库堤坝除险加固工作，建设汀溪至梅山输水工程，推进过芸溪等一批流域综合治理和翔安珩厝东园4．1公里海堤建设，实施1．2万亩生态林、风景林建设。健全农村社会保障体系，全面实施被征地人员基本养老保险，将环东海域退养渔民和造福工程移民纳入参保范围，完善和提高农村合作医疗保险水平。

　　（四）坚持经济特区先行先试，进一步深化改革扩大开放

　　加大改革攻坚力度。继续推进国有企业改革，通过引进战略合作伙伴或转让部分股权等方式，推动国有独资企业投资主体多元化。以提高竞争力和控制力为重点，加强国有资产的监督和管理，积极探索资产—资本—新资产的循环转化机制，防止国有资产流失，促进国有企业做强做大。改革公用事业监管体制和公用设施管养体制，稳步推进文化体制改革综合试点，积极稳妥推进医疗卫生体制改革。深化投融资体制改革，放宽市场准入，鼓励非公有制企业进入公用事业、基础设施、现代服务业等领域，在融资、财税、用地等方面给予扶持。推行财政性工程预选承包商制度，规范项目竣工联合验收制度。

　　集中力量抓好招商引资。充分利用投洽会、台交会、“6?18”项目成果对接会平台，发挥港澳台同胞和华人华侨的作用，坚持内资外资一起抓，大中小项目一起上，劳动、资金、技术“三密集型”项目相配套，着力引进一批高科技含量、高附加值的节能环保型龙头项目，带动关联配套项目。用好内外资企业所得税法合并后的特区过渡期政策，密切关注跟踪国内外大企业投资扩产意愿，加大招商力度，注重结构调整、促进产业升级。引导企业对接境内外行业巨头，通过参股控股、兼并收购等方式做大做强。积极发展服务外包业，争取成为国家重点发展的服务外包业基地城市。

　　积极开拓国际国内市场。落实好出口扶持政策，支持企业到境外设立贸易网点以及参加各类商品展览会、交易会，办好第二届中国（厦门）名牌产品国际采购交易会，扩大汽车及零部件、计算机、液晶电视和光电等优势产品出口。充分发挥保税区、保税物流园区、出口加工区等海关特殊监管区的作用，加强口岸通关、市场开拓、市场预警预测等外贸促进服务体系建设，大力吸引周边货物经厦门口岸进出。制定实施进口扶持政策，培植大宗货物分拨中心，扩大进口规模。集中用好国内市场开拓专项资金，支持企业到产品主销区设立展馆、参加展会，鼓励外资企业加强原材料、零部件本地化配套，提高名牌地产工业品内销市场份额。同时，进一步密切对内区域协作，加强厦漳泉龙城市联盟，密切闽粤赣十三地市的合作，认真做好对口支援、对口帮扶工作。

　　（五）深化对台交流合作，充分发挥前沿平台作用

　　进一步办好台商投资区，积极承接台湾新一波产业转移，争取在光电、软件、信息服务、生物与新医药、汽车电子和动漫产业等方面取得新突破；鼓励现有台资企业在厦门设立总部，进一步开放对台服务贸易，加大力度引进航运物流、医疗教育和中介服务业。鼓励台湾金融机构以多种形式入驻厦门，争取率先开办人民币与新台币计价结算业务。推进对台花卉进出口市场、种苗研发中心、闽台花卉产业高科技园等项目建设，扶持台资农业企业扩大优良品种的引进繁殖，支持涉农行业协会与台湾开展两岸农业交流活动。

　　做大“小三通”，推动海上直航通邮和便捷货运，开展客船带运小件货物业务，争取两岸直航货物在金门转单。继续扩大对金门蔬菜出口，以台湾水果销售集散中心为平台，做大以水果为重点的台湾农产品进口规模。扩大对台、对金门的旅游产业交流对接。建成五通海空联运码头并投入使用，完成和平码头厦金航线业务搬迁，启用国际旅游客运码头。积极创造条件，争取开放大陆居民赴金旅游，争取率先开通赴澎湖、台湾本岛旅游，争取厦门获批成为赴台旅游首批口岸，争取将五年有效《台湾居民来往大陆通行证》的受理范围，由金、马、澎扩大到台湾本岛。推动两岸直航客运和货运包机周末化。

　　发挥闽南文化的特色和优势，加强与台湾的科技、教育、文化、卫生、体育、新闻出版和民间民俗等方面的交流。推进长庚医院和奥林匹克网球中心建设，吸引台湾同胞来厦就医疗养、休闲健身。加强两地青少年、妇女交流交往。继续办好“两岸图书交易会”、“食博会”等涉台展会和第二届“保生慈济文化节”。进一步办好厦门卫视、闽南之声广播、厦门网。全方位、多领域地开展与台中市的交流合作。

　　（六）加快教育卫生文化体育项目建设，促进经济社会协调发展

　　优先发展教育。认真贯彻新修订的《义务教育法》，促进义务教育均衡发展，率先在全省实现“双高普九”和城镇义务教育阶段免收学杂费。认真实施素质教育，加强师资队伍建设，积极稳妥推进高中课改。新建扩建一批中小学校，新增2万个学位，解决好农民工子女就学。开工建设五缘湾学村、集美中学高中部，创办中央音乐学院鼓浪屿钢琴学校，筹建厦门市中学生综合实践基地。制定民办学校管理办法，促进民办教育健康发展。推进厦门理工学院二期、华侨大学集美校区、武汉理工学院厦门校区等项目建设，支持厦门大学新校区开工建设。强化职业技术教育和就业培训，加快高技能人才公共实训基地和高级技工学校新校区建设，培养与厦门产业发展相适应的高素质劳动者。积极拓展华文教育，办好“国际教育展”和“闽台百名校长论坛”。

　　提高卫生事业水平。推进第一医院、妇幼保健院、仙岳医院等扩建工程尽快投入使用，加快中山医院内科大楼、口腔医院迁建等项目前期工作。争创一批三甲医院，加大岛外市级医院建设。建立多种经济成份办医模式，改革和完善公立医院的管理体制和运行机制，确保公立医院的公益性。建设全国一流的医疗急救体系，加强社区卫生服务中心建设，完善城乡医疗卫生服务和医疗救助，力争建成20个标准化村卫生所。办好海峡两岸中医药发展与合作高峰论坛。做好人口与计划生育工作，稳定人口低生育水平，治理出生人口性别比偏高问题，加强流动人口计生管理，提高人口质量，积极应对人口老龄化。

　　促进文化、体育、广播电视事业蓬勃发展。充分发挥文化艺术中心的作用，使之成为真正惠及全市人民的文化艺术活动场所。精心办好第四届中国国际钢琴比赛。做大做强商品油画产业、钢琴业和艺术品拍卖市场，培育闽南语影视音乐制作产业，大力扶持游戏动漫、创意设计等文化产业。启动厦门大剧院前期工作，着手申报鼓浪屿世界文化遗产、“中国南音”世界非物质文化遗产。做好有线数字电视整体转换。建成工人体育馆，推进翔安文体中心、国家乒乓球南方训练基地等项目建设。办好国际马拉松和公路跑协会世界大会，创新和丰富厦门国际马拉松赛内涵，使之成为具有国际影响力的体育赛事，继续推动全民健身。

　　实施人才强市战略。重视现有人才的培养和使用，健全激励机制，真正做到人尽其才，充分发挥人才在经济社会发展中的作用。继续办好各类引才引智活动，拓宽人才资源开发与交流合作渠道，建立完善科学的人才评价制度，加强领军人才的培养与开发，努力吸引高层次人才和急需专门人才。完善人才服务体系，在全社会形成尊重人才、关心人才、重视人才的良好氛围。

　　（七）加强社会管理，夯实构建和谐社会的基础

　　完善就业和社会保障。统筹做好新增劳动力、大中专毕业生、复转军人的就业工作，加强对城乡青壮年无业人员的就业培训和引导，大力开发公益性就业岗位，全年新增就业18万人。加强劳动保障执法监察，设立区级劳动争议仲裁院，建立工资支付保证金制度，维护农民工合法权益。推动企业履行社会责任，完善政府、工会和企业三方协商机制，促进劳动关系和谐。扩大社会保险覆盖面，做好个人账户管理，鼓励多种形式的职工补充养老保险。改革农民工参保办法，全面推进高危行业工伤保险。做好全民医保，巩固新型农村合作医疗，力争农民参保率达到100%。加强社会保险基金监管，保障基金安全。稳步提高最低生活保障标准，完善社会救助体系，发展慈善事业和社会福利事业，重点做好扶老、救孤、助残、济困工作。

　　着力解决人民群众关心的问题。扎实做好十二件为民办实事项目。加快建设社会保障性住房，新开工保障性住房2．1万套，严格保障性住房分配和管理，保证公开公平公正。扩大社会保障卡在医疗领域的运用，开通市民健康信息系统，规范医疗服务收费项目，努力解决群众看病难、看病贵问题。重视粮食储备与供应工作，全面强化食品药品、餐饮卫生监管，严厉打击制售假冒伪劣行为，保障市民用药进食安全放心。优先发展城市公共交通，加快公交场站和农村客运站建设，优化公交线路布局，提高公交服务质量。

　　强力构筑“平安厦门”。全面落实社会治安综合治理责任制，推进社会治安基层基础工程建设，完善社会治安防控体系，实施科技强警，加强警务改革，充实一线警力。深入开展打黑除恶、缉枪治爆、扫黄打非、禁毒禁赌等工作。加大反洗钱工作力度，完善金融风险防范和处置机制。加强应急管理体制机制和预案体系建设，提高公共安全保障和处置突发事件能力。加强信访工作，完善社会舆情汇集分析机制和矛盾纠纷排查调处机制，预防和控制群体性事件。落实各级政府安全生产监管责任制，严格安全生产许可制度，建立健全重大危险源安全监控体系，重点抓好海陆交通、危险化学品、非煤矿山、建筑施工安全专项整治，严肃查处事故责任，努力营造安全的社会环境。

　　做好新一轮文明城市创建工作。按照建设现代化、国际化文明城市目标，适应新的文明城市测评体系，建立和完善文明创建长效机制。加大基层社区投入力度，理顺社区管理体制，充实加强基层力量，完善社区管理服务，扎实推进和谐社区和文明村镇建设。加强社会主义核心价值体系建设，深入实践以“八荣八耻”为主要内容的社会主义荣辱观，贯彻落实《公民道德建设实施纲要》，突出抓好未成年人思想道德建设和大学生思想政治工作。加强城市信用体系建设，推进政务诚信、商务诚信和社会诚信建设。充分发挥工会、共青团、妇联等群团组织以及各类行业协会的作用，切实做好民族宗教、外事侨务工作，加强人防海防及国防交通建设。完善国防动员机制，积极构建优抚安置工作新机制，深入开展双拥活动，进一步关心支持驻厦部队建设，加强军民军政团结，争创“全国双拥模范城市”六连冠。

　　三、加快政府职能转变，推进政府管理创新

　　扎实推进新一轮跨越式发展，必须以改革创新为动力，以提高执行力、公信力为重点，坚持不懈地推进政府自身建设。

　　强化工作落实。各级政府领导和工作人员要积极适应国际形势变化和国家重大政策调整，把落实科学发展观和构建和谐社会贯穿到政府工作的各个方面，用良好的政风扎实推进新一轮大发展。坚持实事求是、求真务实，讲实话、出实招、办实事、重实效，重视基础工作和基层建设，把更多的时间和精力用在调查研究上，用在帮助解决基层和群众的实际问题上。建立健全科学的政府绩效评估体系和经济社会发展综合评价体系，进一步改进工作作风，精简会议、文件，减少各类评比达标活动，促使各项决策和工作部署落到实处，取得实效。

　　推进管理创新。更加注重履行社会管理和公共服务职能，优化机构设置，理顺政府部门分工，完善市区管理体制和社会管理方式，寓管理于服务之中，充分利用社会资源，促进社会自治管理，努力实现政府调控机制同社会协调机制互联、政府行政功能同社会自治功能互补、政府管理力量同社会调节力量互动。参照国际惯例和先进城市做法，深化行政审批制度改革，最大限度简化手续、缩短时限。要加快电子政务建设，改进跨部门的电子政务系统，扩大网上并联审批。要进一步加强业务学习，不断提高驾驭经济社会工作的本领、破解发展难题的本领和做好群众工作的本领，善于捕捉国际信息，创新工作思路，善于用改革的思路、发展的办法、市场经济的手段，不断解决深层次的矛盾和问题，使政府各项工作体现时代性、把握规律性、富于创造性。

　　坚持依法行政。认真执行人大及其常委会的各项决议、决定，依法接受市人大及其常委会的法律监督、工作监督，自觉接受市政协的民主监督，定期向市人大及其常委会报告工作和向市政协通报情况，认真做好人大代表议案、建议和政协委员提案的办理工作，健全与民主党派、工商联、无党派人士的联系制度。重视新闻舆论和社会公众监督，支持监察、审计部门依法独立监督，增强行政透明度。完善政府公报、政府网站、政府新闻发布会等公开渠道，深入推动政务公开。继续落实《行政许可法》，健全规范性文件法律审查制度，及时废止、修订不合时宜的各项规定。认真贯彻《监督法》，全面推行行政执法责任制，加强行政执法监督，完善行政执法评议考核、行政赔偿和过错追究制度，最大限度减少行政执法自由裁量权，做到用权受监督、侵权要赔偿、违法必追究。深入开展“五五”普法教育，在公务员队伍中形成学法、守法、依法行政的良好氛围。

　　做到清正廉明。落实“两个务必”，执行“八个坚持、八个反对”，大力倡导“八个方面”的良好风气，坚持不懈地加强廉政建设，落实“一岗双责”，增强忧患意识、公仆意识、节俭意识，坚定不移地开展反腐斗争，推进教育、制度、监督并重的惩治和预防腐败体系建设。加强廉政文化建设，丰富廉政教育的形式和方法，使正面教育更有感染力、反面教育更有警示力、自我教育更有律己性、经常教育更有针对性。加大力度从源头上防治腐败，推广实施“阳光工程”，认真开展治理商业贿赂专项工作，严肃查办虚假招投标、非法批地、擅自变更规划设计获取利益等案件，继续整治教育乱收费，纠正医药购销和医疗服务中的不正之风。加大对财政性投资的监督管理力度，严格控制公务消费。在严肃惩处违纪违法行为的同时，对实事求是、坚持原则、秉公办事、开拓创新的干部给予支持保护和鼓励。充分发挥全体公务员的积极性和创造性，致力提升公务人员素质和凝聚力，建设一个行为规范、勇于负责、公正透明、清正廉洁、勤政高效、务实创新，人民群众满意的政府。

　　各位代表！

　　让我们更加紧密地团结在以胡锦涛同志为总书记的党中央周围，高举邓小平理论和“三个代表”重要思想伟大旗帜，全面落实科学发展观，加快构建社会主义和谐社会，在中共厦门市委的坚强领导下，同心协力，锐意进取，开拓创新，再造辉煌，实现经济社会又好又快发展，以优异成绩迎接党的十七大胜利召开！
